# Response to the Letter by Matsubara

**DOI:** 10.31662/jmaj.2024-0420

**Published:** 2025-03-07

**Authors:** Masaki Mori

**Affiliations:** 1 Tokai University School of Medicine, Isehara, Japan

**Keywords:** artificial intelligence, medicine, machine learning, deep learning, neural network

We appreciate and agree with the comments by Dr. Matsubara that real-world evidence is needed to establish the benefit versus risk of using artificial intelligence (AI) models in writing assistance ^(1)^. We also agree that the method proposed by Dr. Matsubara would be a good strategy to identify the impact of using AI language models.

Dr. Matsubara also raised an important point about clarifying the use of AI for writing assistance even in the case of permitting the use. We think that whenever using writing assistance with AI, it is important to include the following information (1) which AI was used, (2) what version was used, (3) what prompts were used and (4) which part was assisted by AI. This information is important to ensure that publishers and researchers can identify the cause in case of any problem (such as those caused by hallucinations ^(2)^ and adverse attacks, which are still a frequent problem in AI) ^(3), (4)^.

As Dr Matsubara states, the difficulty of this experiment is in its implementation. We totally agree with his point that which journal will take the initiative will be a challenge. In addition to these aspects, the confidentiality of unpublished articles may become a problem. Most AI models are known to use the input data to further improve the AI ^(5)^. This means that the information may become publicly available through the AI before being published in a journal. However, the impact of this fact may have decreased recently since it is increasingly becoming normal to allow pre-prints before publishing the reviewed article.

All of these discussion supports the lack of definitive evidence on how to regulate the use of AI in writing. Further research in this area is urgently needed for this emerging technology.

## Article Information

### Conflicts of Interest

None

### Disclaimer

Masaki Mori is one of the Associate Editors of JMA Journal and on the journal’s Editorial Staff. He was not involved in the editorial evaluation or decision to accept this article for publication at all.
